# Characteristics of Adults With Addictions and Mental Health Problems Who Have Experienced Homelessness: A Population-Based Study From Alberta, Canada: Caractéristiques des adultes aux prises avec des problèmes de dépendance et de santé mentale et ayant connu l’itinérance : une étude fondée sur la population de l’Alberta, Canada

**DOI:** 10.1177/07067437251380732

**Published:** 2025-10-08

**Authors:** Rebeca Barry, Geoffrey Messier, Anees Bahji, Gina Dimitropoulos, Sumantra Monty Ghosh, Julia Kirkham, Scott B Patten, Katherine Rittenbach, Faezehsadat Shahidi, David Tano, Valerie H Taylor, Dallas P Seitz

**Affiliations:** 1 Department of Psychiatry, 173447 University of Calgary, Calgary, AB, Canada; 2 Hotchkiss Brain Institute, 173447 University of Calgary, Calgary, AB, Canada; 3 550962 Mathison Centre for Mental Health Research and Education, 173447 University of Calgary, Calgary, AB, Canada; 4 Department of Electrical and Software Engineering, 2129 University of Calgary, Calgary, AB, Canada; 5 Department of Community Health Sciences, 173447 University of Calgary, Calgary, AB, Canada; 6 Faculty of Social Work, 173447 University of Calgary, Calgary, AB, Canada; 7 70401 Cumming School of Medicine, 173447 University of Calgary, Calgary, AB, Canada

**Keywords:** homelessness, health services, mental disorders, multimorbidity

## Abstract

**Objective:**

This study seeks to understand the characteristics of individuals with addictions and other mental health (AMH) conditions who had a history of homelessness compared to those who did not experience homelessness.

**Method:**

This cross-sectional analysis used linked administrative data from Alberta, Canada on April 1, 2018. People with AMH who experienced homelessness in the year prior to index were identified using hospitalisations and emergency department (ED) visits. We used multivariable logistic regression to evaluate the association between a set of descriptive variables and homelessness, adjusted for age and sex.

**Results:**

Among the 622,614 individuals with AMH conditions, 3,390 (0.54%) had an indicator of homelessness. People experiencing homelessness (PEH) were younger (mean = 39 vs. 42 years, *p* < .001) and more frequently male (66% vs. 41%, *p* < .001) than individuals not experiencing homelessness. PEH were also more likely to be diagnosed with multiple AMH disorders (44.8% diagnosed with ≥ 4 AMH conditions vs. 3.8% of individuals without homelessness). PEH were more likely to have a history of visiting a psychiatrist (adjusted odds ratio (AOR) = 8.11, 95% CI [7.47–8.80], having an ED visit for AMH reasons (AOR = 25.44, 95% CI [22.94–28.21], and to have been hospitalised for AMH reasons (AOR = 13.53, 95%CI [12.61–14.52]).

**Conclusions:**

Within the population of individuals with diagnosed AMH conditions, PEH demonstrated increased AMH complexity, greater healthcare utilisation and a greater likelihood of almost all AMH disorders. Given the complex mental health needs of this group, they will require more intensive mental health and general medical services that must be integrated with housing and additional social support systems.

## Résumé

**Objectif:**

Cette étude vise à comprendre les caractéristiques des personnes aux prises avec une dépendance et d’autres problèmes de santé mentale qui avaient des antécédents d’itinérance comparativement à celles qui n’avaient pas connu l’itinérance.

**Méthode:**

Cette analyse transversale a été réalisée à l’aide de données administratives liées entre elles recueillies en Alberta, au Canada, le 1^er^ avril 2018. Les personnes aux prises avec une dépendance et d’autres problèmes de santé mentale et ayant connu l’itinérance au cours de l’année précédant la date de référence ont été repérées à l’aide de leurs hospitalisations et de leurs visites au service des urgences. Nous avons utilisé la régression logistique multivariable pour évaluer l’association entre un ensemble de variables descriptives et l’itinérance, ajustée en fonction de l’âge et du sexe.

**Résultats:**

Parmi les 622 614 personnes aux prises avec une dépendance et d’autres problèmes de santé mentale, 3 390 (0,54%) avaient un indicateur d’itinérance. Les personnes ayant vécu l’itinérance étaient plus jeunes (moyenne = 39 ans contre 42 ans, *p* < 0,001) et plus souvent des hommes (66% contre 41%, *p* < 0,001), par rapport aux personnes n’ayant pas vécu l’itinérance. Les personnes ayant vécu l’itinérance étaient également davantage susceptibles d’être diagnostiquées avec de multiples troubles de dépendance et de santé mentale (44,8% ont reçu un diagnostic ≥ 4 troubles de dépendance et de santé mentale contre 3,8% des personnes n’ayant pas vécu l’itinérance). Les personnes ayant vécu l’itinérance étaient davantage susceptibles d’avoir des antécédents de consultation psychiatrique (rapport de cotes ajusté = 8,11 [IC à 95% = 7,47 à 8,80]), de consulter au service des urgences en raison de troubles de dépendance et de santé mentale (rapport de cotes ajusté = 25,44 [IC à 95% = 22,94 à 28,21]) et d’avoir été hospitalisées en raison de troubles de dépendance et de santé mentale (rapport de cotes ajusté = 13,53 [IC à 95% = 12,61 à 14,52]).

**Conclusions:**

Au sein de la population de personnes chez qui des troubles de dépendance et de santé mentale ont été diagnostiqués, les personnes ayant vécu l’itinérance ont présenté des troubles de dépendance et de santé mentale d’une plus grande complexité, un recours plus important aux soins de santé et une plus grande probabilité de souffrir de presque tous les troubles de dépendance et de santé mentale. Compte tenu des besoins complexes en santé mentale de ce groupe, il sera nécessaire de lui proposer des services de santé mentale et de médecine généraliste plus intensifs qui doivent être intégrés à des systèmes de logement et d’assistance sociale supplémentaires.

## Introduction

Addictions and other mental health (AMH) conditions present significant social and economic burdens.^1^ Globally, people experiencing homelessness (PEH) have a high prevalence of AMH conditions, with approximately 67% diagnosed with a current AMH condition.^2^ When examining specific disorders among PEH, a high prevalence of substance use disorders (44%), major depression (19%), schizophrenia (7%), and bipolar disorder (8%) have been identified among PEH.^2^ Despite the high prevalence of AMH among PEH, they face several barriers to AMH services including stigma, poor integration of AMH and housing programmes, and financial barriers to care.^3,4^ PEH may encounter not only increased health challenges, but also heightened social and legal complexities. For instance, a study conducted in Toronto, Canada found that among PEH with AMH conditions, a relatively high proportion also had interactions with police.^5^

While there is known to be a high prevalence of AMH among PEH, to our knowledge, there are few large-scale population-based studies which have examined the characteristics (in particular, health-care utilisation and AMH multimorbidity) of individuals diagnosed with AMH who experience homelessness compared to other individuals diagnosed with AMH conditions.^6^ Most studies employ a study population consisting exclusively of PEH,^2^ likely due to challenges with ascertaining a larger population-based comparison group. This study adopts a novel approach by utilising health administrative data to include a meaningful comparison group of individuals diagnosed with AMH conditions. Understanding the prevalence and characteristics of individuals experiencing homelessness among people diagnosed with AMH is a necessary first step towards examining the complex relationship between AMH and homelessness and to better understand the unique health needs of this population.

This study aims to compare the prevalence and characteristics of AMH conditions and health-care utilisation of individuals with AMH conditions who experienced recent homelessness compared to those who did not. Our findings will help inform the need for comprehensive and intensive mental health and general medical services that are integrated with housing and other social support systems.

## Methods

This cross-sectional analysis utilises linked data from April 1, 2013 until April 15, 2018. Data was extracted from the Alberta Health Services (AHS) Enterprise Data Warehouse with support provided by the Alberta Strategy for Patient Oriented Research Support Unit (AbSPORU).

### Data Sources

This analysis uses health services data from AHS, Alberta Health and associated administrative data. Our data sources include the Physician Claims database, the Canadian Institute for Health Information Discharge Abstract Database (CIHI-DAD), the CIHI National Ambulatory Care Reporting System (CIHI-NACRS), the Registry database, the Pampalon Material and Social Index Deprivation database, and Vital Statistics. These datasets were linked using unique encoded identifiers based on de-identified personal health numbers.

### Inclusion Criteria

We included all individuals diagnosed with an AMH condition within 5 years prior to the index date of April 1, 2018 living in Alberta, Canada. AMH conditions were defined based on either one hospitalisation with a relevant AMH diagnosis or two relevant AMH diagnosis claims at least 30 days apart within a 2 year time period. We used any recorded code in DAD or claims, and did not limit AMH diagnoses to only primary diagnoses. All relevant AMH diagnoses codes are documented in Table 1. We categorised AMH disorders into 15 groups as follows: mood disorders, anxiety disorders, substance use disorders (excluding tobacco or nicotine dependence), psychotic disorders, cognitive disorders, developmental disabilities, personality disorders, eating disorders, sexual disorders, attention-deficit/hyperactivity disorder, other childhood and developmental disorders, organic disorders, sleep disorders, somatic disorders and self-harm. Where algorithms were available, codes were based on validated algorithms.^7–13^ Individuals were aged 18 to 65 and alive on the index date. Individuals had to be included in the provincial health insurance registry on the index date.

### Case Definition for Homelessness

PEH over the past year were identified through hospitalisations (using DAD) or emergency department (ED) visits (using NACRS) with diagnostic codes Z59.0 or Z59.1.^14^ The time period for the definition of homelessness was the year prior to the index date of April 1, 2018 with an additional 15 days before and after this time period (assessment period: March 15, 2017–April 15, 2018), meeting the validated algorithm criteria for “annual homelessness.”^14^ This definition of homelessness has a specificity of 99.9%, with a sensitivity ranging from 19–36%.^14^

### Factors Potentially Associated With Homelessness

We examined several categories of variables potentially related to homelessness including demographics, psychiatric multimorbidity, general medical conditions, health encounters involving police and healthcare utilisation.

*Demographics:* We examined demographic information including age and sex. We used the Registry data, a population-based registry for the province of Alberta which includes data on individual's age and sex and eligibility for public health care insurance. We used the material and social Pampalon deprivation indices^15^ to examine socioeconomic status. These are area-based measures of deprivation, based on census data for most recent postal codes.

*Psychiatric multimorbidity and dual diagnosis:* Multimorbidity refers to the presence of multiple health conditions, with psychiatric multimorbidity referring to the presence of multiple psychiatric conditions.^16^ Dual diagnosis refers to the presence of both a substance use disorder and another mental health disorder.^16^ We examined psychiatric multimorbidity by estimating the occurrence of two or more types of psychiatric conditions (of the 15 categories defined above). We examine the prevalence of dual diagnosis by examining the prevalence of both a substance use disorder and other mental health diagnosis.

*General medical comorbidity and multimorbidity:* We use the Elixhauser comorbidity^17,18^ index, based on Physicians Claims data, CIHI-DAD and CIHI-NACRS to measure general medical comorbidities. We also examined the prevalence of traumatic brain injury. We examined general medical multimorbidity by estimating the prevalence of those with two or more Elixhauser comorbidities, excluding mental health related comorbidities.

*Health encounters involving police:* Health encounters involving police services are defined as any DAD or NACRS ICD-10 diagnostic code of Y35.0–Y35.7, Z65.0–Z65.3, or any claims diagnostic code of E970–E976, E978, or V625. Health encounters involving police services are not capturing community-level police interactions, but solely interactions that lead to an ED or hospitalisation with a relevant code.

*Health-care utilisation:* We examined ED visits using CIHI-NACRS. We examined physician visits using the Physicians Claims database. We examined hospitalisations using CIHI-DAD.

### Statistical Analysis

To compare the baseline characteristics of PEH compared to individuals who did not experience homelessness, we used t-tests to compare means, chi-square tests to compare proportions, and the Wilcoxon Rank-Sum test to compare medians. Multiple logistic regression models were used to determine the association between each baseline characteristics and homelessness using age and sex adjusted odds ratios (ORs). Interaction terms by sex were explored for all logistic regression models to determine if the associations varied between sexes. Analyses were completed using SAS version 9.4^19^ using two-sided *p*-values of .05 as the threshold for statistical significance.

### Sensitivity Analyses

A sensitivity analysis was performed, where we extended the definition of homelessness to also include the ICD-9 claims codes V60.0 and V60.1 in the year prior to index, with the additional 15 days before and after to mirror the ascertainment window for the previously defined homelessness variable (March 16, 2017–April 15, 2018). A second sensitivity analysis was conducted where only those with an emergency room visit or hospitalisation in the year prior to index were included in the study population (with an additional 15 days before and after).

### Ethics

This study was reviewed and approved by the Conjoint Health Research Ethics Board at the University of Calgary (REB21-0070).

## Results

Among the 662,614 individuals meeting study criteria, 3,390 (0.54%) were identified as having experienced homelessness over the year prior to index. PEH were marginally younger (mean age = 39 vs. 41 years, *p* < .001) and more likely to be male (66% vs. 41%, *p* < .001) than individuals with AMH conditions not experiencing homelessness (Table 1). PEH also scored higher on both the social and material Pampalon deprivation indices (*p* < .001), indicating greater deprivation. While the interaction terms by sex were significant in some models, the overall direction and significance of the associations were consistent among males and females, so results were reported among the entire population.

**Table 1.**
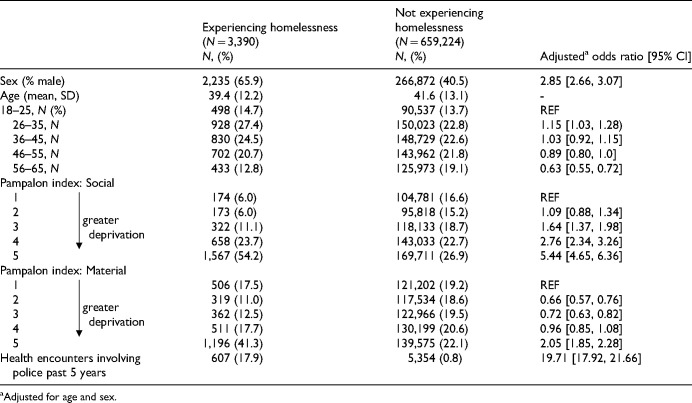
Baseline Characteristics Among People Who Did and Did Not Experience Homelessness.

Among the entire study population, the most common AMH conditions were anxiety disorders (68.6%), mood disorders (49.6%), and substance use disorders (11.6%). Among PEH, the most common disorders were also substance use disorders (82.1%), anxiety disorders (77.0%) and mood disorders (73.3%) (Table 2). In the 5 years prior to index, after adjusting for age and sex, PEH were more likely to be diagnosed with all AMH disorders other than sleep disorders and sexual disorders (Table 2). Most notably, PEH were more likely to be diagnosed with substance use disorders (AOR = 32.74, 95% CI [29.93–35.81]), psychotic disorders (AOR = 10.99, 95% CI [10.21–11.83]), personality disorders (AOR = 12.67, 95% CI [11.77, 13.65]), and to have an incident of self-harm (AOR = 9.87, 95% CI [8.62–11.31]) when compared to people not experiencing homelessness.

**Table 2.** AMH Diagnoses in Past 5 Years Among People Who Did and Did Not Experience Homelessness.

Disorder	Experiencing homelessness (*N* = 3,390)	Not experiencing homelessness (*N* = 659,224)	Adjusted^a^ odds ratio [95% CI]	*p*-value
	*N*, (%)	*N*, (%)		
Anxiety disorder	2,610 (77.0)	453,428 (68.8)	1.79 [1.65, 1.94]	<.001
Mood disorder	2,484 (73.3)	319,285 (48.4)	3.20 [2.97, 3.46]	<.001
Substance use disorder	2,783 (82.1)	74,189 (11.3)	32.74 [29.93, 35.81]	<.001
Psychotic disorder	1,132 (33.4)	25,089 (3.8)	10.99 [10.21, 11.83]	<.001
Cognitive disorder	67 (2.0)	5,117 (0.8)	2.90 [2.27, 3.71]	<.001
Developmental disability	119 (3.5)	6,360 (1.0)	2.86 [2.37, 3.45]	<.001
Personality disorder	1,115 (32.9)	23,210 (3.5)	12.67 [11.77, 13.65]	<.001
Eating disorder	16 (0.5)	1,902 (0.3)	2.41 [1.47, 3.95]	.047
Sexual disorder	50 (1.5)	15,469 (2.4)	0.43 [0.32, 0.57]	.003
Other childhood and developmental disorders	190 (5.6)	9,423 (1.4)	3.71 [3.19, 4.32]	<.001
Attention deficit hyperactivity disorder	325 (9.6)	40,458 (6.1)	1.24 [1.10, 1.39]	<.001
Organic disorders	237 (7.0)	9,295 (1.4)	5.05 [4.41, 5.77]	<.001
Sleep disorders	160 (4.7)	30,010 (4.6)	0.96 [0.82, 1.13]	.641
Somatic symptoms and related disorders	67 (2.0)	7,332 (1.1)	1.85 [1.45, 2.36]	<.001
Self-harm	240 (7.1)	4,950 (0.8)	9.87 [8.62, 11.31]	<.001

*Note.* AMH = addictions and other mental health.

^a^ Adjusted for age and sex.

When examining psychiatric multimorbidity, PEH had greater number of AMH conditions (median = 3 vs. 1, *p* < .001), and greater proportion had multiple AMH conditions, with 44.8% of PEH diagnosed with four or more types of AMH conditions compared to only 3.8% of individuals who did not experience homelessness (*p* < .001). Dual diagnosis was identified in 72.6% of PEH compared to only 8.2% of people not experiencing homelessness. Psychiatric multimorbidity was present 84% of PEH versus 38.5% of people not experiencing homelessness.

When examining general medical conditions based on the Elixhauser Comorbidity Index as shown in Table 3, PEH were more likely to be diagnosed with most chronic health conditions including chronic obstructive pulmonary disorder, hypertension, and congestive heart failure. 52.8% of PEH met the criteria for general medical multimorbidity versus only 26.6% of people without a history of homelessness (Table 4).

**Table 3.** General Medical Conditions Among People Who Did and Did Not Experience Homelessness.

Condition	Experiencing homelessness (*N* = 3,390)	Not experiencing homelessness (*N* = 659,224)	
	N, (%)	N, (%)	*p*-value
Mean Elixhauser score^a^ (SD)	2.21 (2.16)	1.08 (1.35)	<.001
Congestive heart failure	182 (5.4)	12,326 (1.9)	<.001
Cardiac arrhythmia	180 (5.3)	8,154 (1.2)	<.001
Hypertension, uncomplicated	907 (26.8)	164,745 (25.0)	.018
Hypertension, complicated	24 (0.7)	2,655 (0.4)	.005
Other neurological disorders	610 (18.0)	28,578 (4.3)	<.001
Chronic obstructive pulmonary disease	1,230 (36.3)	155,242 (23.6)	<.001
Diabetes, uncomplicated	211 (6.2)	13,389 (2.0)	<.001
Diabetes, complicated	154 (4.5)	9,101 (1.4)	<.001
Liver disease	634 (18.7)	21,233 (3.2)	<.001
Peptic ulcer disease	45 (1.3)	1,178 (0.2)	<.001
AIDS/HIV	118 (3.5)	1,961 (0.3)	<.001
Anemia	38 (1.1)	992 (0.2)	<.001
Traumatic brain injury/head injury	415 (12.2)	6,819 (1.0)	<.001

*Note.* AMH = addictions and other mental health.

^a^ Excluding AMH conditions.

**Table 4.** Number of AMH Categories at Baseline Among People Who Did and Did Not Experience Homelessness.

	Experiencing homelessness (*N* = 3,390)	Not experiencing homelessness (*N* = 659,224)	
Disorder	*N*, (%)	*N*, (%)	*p*-value
One category of AMH disorder^a^	541 (16.0)	405,629 (61.5)	<.001
Two categories of AMH disorders	553 (16.3)	178,727 (27.1)	<.001
Three categories of AMH disorders	778 (23.0)	49,998 (7.6)	<.001
Four or more categories of AMH disorders	1,518 (44.8)	24,870 (3.8)	<.001
Median number of AMH conditions, IQR	3 (2, 5)	1 (1, 2)	<.001
Dual diagnosis	2,462 (72.6)	54,088 (8.2)	<.001
Psychiatric multimorbidity	2,849 (84.0)	253,955 (38.5)	<.001
General medical multimorbidity	1,789 (52.8)	175,345 (26.6)	<.001

^a^ There are 15 categories of AMH conditions, as categorised in Table 2.

When examining health service use for mental health reasons, in the 5 years leading to the index date, PEH were more likely to visit EDs for mental health reasons (AOR = 25.44, 95% CI [22.94–28.21]) (Table 5). They are also more likely to visit a psychiatrist (AOR = 8.11, 95% CI [7.47–8.80]). PEH were also more likely to be hospitalised for mental health reasons (AOR = 13.53, 95% CI [12.61–14.52]).

**Table 5.** Healthcare Utilisation Among People Who Did and Did Not Experience Homelessness.

Utilisation	Experiencing homelessness (*N* = 3,390)	Not experiencing homelessness (*N* = 659,224)	Adjusted odds ratio [95% CI]*
	*N*, (%)	*N*, (%)	
Physician visits (claims)			
Any family physician visit (1 year)	3,178 (93.8)	609,942 (92.5)	1.56 [1.36, 1.79]
Any family physician visit (5 year)	Suppressed (∼100)	657,794 (99.8)	Suppressed
Any psychiatrist visit (1 year)	2,096 (61.8)	96,293 (14.6)	8.92 [8.32, 9.57]
Any psychiatrist visit (5 year)	2,652 (78.2)	193,733 (29.4)	8.11 [7.47, 8.80]
Any other specialist visit (1 year)	1,705 (50.3)	304,007 (46.1)	1.42 [1.33, 1.52]
Any other specialist visit (5 year)	2,730 (80.5)	531,104 (80.6)	1.27 [1.16, 1.38]
Emergency visits (NACRS)			
Any ER visit (1 year)	3,349 (98.8)	360,069 (54.6)	76.54 [56.25, 104.16]
Any ER visit (5 year)	Suppressed (∼100)	587,979 (89.2)	158.54 [51.24, 490.50]
Any ER mental health visit (1 year)	2,487 (73.4)	49,035 (7.4)	33.58 [31.07, 36.29]
Any ER mental health visit (5 year)	2,975 (87.8)	142,870 (21.7)	25.44 [22.94, 28.21]
Any non-mental health ER visits (1 year)	3,083 (90.9)	343,149 (52.1)	10.63 [9.45, 11.96]
Any non-mental health ER visits (5 year)	3,343 (98.6)	578,075 (87.7)	11.85 [8.89, 15.81]
Hospitalisations (DAD)			
Any hospitalisation (1 year)	2,040 (60.2)	33,587 (5.1)	27.68 [25.81, 29.69]
Any hospitalisation (5 year)	2,711 (80.0)	116,732 (17.7)	18.20 [16.72, 19.80]
Any mental health hospitalisation (1 year)	953 (28.1)	8,345 (1.3)	27.90 [25.76, 30.21]
Any mental health hospitalisation (5 year)	1,412 (41.7)	30,716 (4.7)	13.53 [12.61, 14.52]

*Note.* NACRS = National Ambulatory Care Reporting System; DAD = Discharge Abstract Database.

* Adjusted for age and sex.

When examining other health service usage, PEH were also more likely to visit ERs for non-mental health (AOR = 11.85, 95% CI [8.89–15.81]) reasons (Table 5). PEH were also more likely to be hospitalised (AOR = 18.20, 95% CI [16.72–19.80]). Finally, PEH were also more likely to have healthcare encounters involving police services (17.9% vs. 0.8%, *p* < .001).

The sensitivity analysis expanding the definition of homelessness classified an additional 561 individuals as experiencing homelessness, for a total of 3,951 PEH. The results of the sensitivity analysis (eTables 3–6) revealed similar findings to the original analysis. The second sensitivity analysis limiting the study population to only those with a DAD or NACRS record in the same time period required to meet the homelessness definition resulted in a study population of 376,927. This analysis revealed similar findings to the original analysis, except both PEH and people not experiencing homelessness were similarly likely to visit EDs.

## Discussion

Our study indicates that among individuals diagnosed with AMH, those who experienced homelessness had greater mental health and general medical multimorbidity, a history of increased mental health and general medical healthcare utilisation compared to people who did not experience homelessness. They are more likely to meet the criteria for dual diagnoses. They are also younger, more likely to be male, and more likely to live in areas of greater social and material deprivation.

Several AMH conditions had markedly increased prevalence among PEH including substance use disorders, psychotic disorders, personality disorders and incidents of self-harm. This is consistent with previous research indicating that these disorders may be much more common among PEH.^2,20^ Recognition of the pervasiveness of these disorders among PEH should lead to more tailored treatment options and housing options that recognise and overcome the challenges that each of these disorders presents.

Individuals diagnosed with AMH conditions often have significant health needs and these needs are further elevated among PEH. There is evidence that PEH may have a higher prevalence of chronic diseases compared to both the general population and people living in deprived areas based on income, employment, education and other measures.^21^ However, PEH experience barriers to accessing primary care such as competing needs (e.g., housing, income, and food) and difficulty accessing transportation.^22^ Furthermore, previous research has also identified barriers to receiving good general medical healthcare among people diagnosed with severe AMH conditions, such as lack of access to and high costs of integrated care, stigmatisation of patients with AMH conditions, physical complaints regarded as psychosomatic symptoms by practitioners, and difficulties communicating physical symptoms.^23^

While it is known that multimorbidity within mental health disorders is pervasive,^24^ our study indicates that PEH are at much higher risk of being diagnosed with psychiatric and general medical multimorbidity. As co-occurring disorders may represent an interaction among risk factors,^25^ it is possibly that the interaction between homelessness and other risk factors may greatly increase the risk of multimorbidity. Psychiatric multimorbidity requires treatment models of care that integrate co-occurring disorders.^25^ Therefore, PEH may experience complex treatment challenges that may not be adequately addressed by single interventions or individual clinicians.^26^ This may be reflected in the relatively higher healthcare utilisation we observed among PEH. Instead, collaborative care models incorporating evidence-based integrative medicine may be more appropriate for this population.^26^ Interventions are needed to both reduce symptom severity among PEH and to reduce risk of homelessness among people diagnosed with AMH conditions.

Finally, although we found that 17.9% of PEH with AMH conditions had health encounters that involve police, this is a notably lesser proportion than the 55.8% of PEH with AMH that were found to have general community-based police interactions in Toronto, Canada.^5^ Therefore, healthcare encounters involving police may not be representative of general police interactions. Future research may want to investigate the proportion of PEH with AMH conditions with police involvement using data from police services which was not available in the current study.

The increased healthcare utilisation found among PEH may be partially explained by the higher proportion of those with psychiatric and general medical multimorbidity. It may also reflect greater frequency or severity of symptoms. It has been shown that accessing ED services is often indicative of difficulty accessing other more appropriate forms of healthcare within the community.^27^ Therefore, the significantly higher number of both mental health and non-mental health ED visits among PEH may indicate poorer healthcare access among this population. Observational studies are needed to examine potential causal relationships and potential mediators of the relationship between homelessness and healthcare access and utilisation. The relationship between homelessness and these baseline characteristics may be bidirectional. For example, homelessness may exacerbate comorbid conditions, while comorbid conditions may lead to increased risk of homelessness.^28^ It is also possible that there are common factors that may increase risk for both homelessness and AMH conditions.^29^

Several evidence-based strategies can be used to improve care for people diagnosed with AMH who are experiencing homelessness. Housing first strategies provide housing initially without any preconditions, and then later aim to incorporate other supports.^30,31^ Critical time interventions connect individuals to mental health supports during specific timeframes, such as transitions from shelters or inpatient psychiatric care.^30,32^ While both housing first and critical time interventions improve housing retention, their impact on AMH conditions is mixed.^32–34^ Assertive community treatment, involving a multidisciplinary team providing intensive community-based mental health services, has demonstrated effectiveness in reducing AMH symptom severity and homelessness among people with AMH conditions.^35^ However, more evidence-based methods that incorporate collaborative care models and evidence-based integrative care are needed to lower AMH risk and severity among those experiencing or at risk of homelessness.^26,36^

### Strengths and Limitations

First, it is notably difficult to ascertain homelessness using administrative data, as the definition we used for homelessness has an estimated sensitivity of only 19–36%.^14^ Based on the low sensitivity and very high specificity (99.9%), we expect that a number of people experiencing homelessness are incorrectly classified as not experiencing homelessness. The impact on our findings depends on whether the individuals who are correctly classified as experiencing homelessness differ in terms of the characteristics we examined from those who were misclassified as housed. It is also possible that this algorithm may be less likely to capture people experiencing so-called hidden homelessness, such as those living with friends or in their vehicle.^37^ Second, the definition of homelessness necessitates an ED visit or hospitalisation in the year prior to index, which may result in a misclassification bias where only individuals with more severe symptoms or higher healthcare utilisation may be assessed as meeting the criteria for homelessness. It is also known that PEH may avoid healthcare services due to stigmatisation and other barriers.^38^ Our first sensitivity analysis included claims codes in addition to the hospitalisation and ED visit codes to assess for homelessness, to help control for severity of symptoms and healthcare utilisation. However, these codes are also infrequently used and likely also have a low sensitivity. This study is limited by its reliance on healthcare data, which may bias results toward individuals with higher healthcare utilisation, potentially impacting generalizability to PEH who are less likely to engage with healthcare services. Furthermore, we can only capture the diagnosed prevalence of a given condition which consistently underestimates the true prevalence of mental health conditions, particularly among common disorders such as mood disorders.^39,40^ The underestimation may also be different between the two groups. Another limitation is that although where possible we used validated definitions, some algorithms were not validated. For example, to our knowledge there is no validated algorithm for health encounters that involve police so instead we used all police-related diagnostic codes we deemed relevant. Additionally, these codes capture solely police interactions that occur within healthcare settings; they do not account for any police encounters outside healthcare contexts. The data used in our study are from an earlier time period which may not reflect current characteristics of individuals with addiction and mental health problems who experienced homelessness. Including these earlier study dates was necessary to allow sufficient duration of follow-up in which to examine health outcomes associated with homelessness in the subsequent objectives of our research project. Finally, as this is a cross-sectional study, causality cannot be established. Future longitudinal studies are needed to explore the potential bidirectional relationship between homelessness and AMH conditions.

This study has strengths. First, our administrative data gives us access to all individuals with diagnosed AMH conditions in Alberta. This gives us access to a large study population with the ability to detect smaller differences between groups. Given that all individuals living in Alberta who have ever accessed healthcare in Alberta were screened for inclusion, this study is less prone to selection bias than a survey or other study method. In this study, all recorded diagnostic codes are used, reducing the likelihood of misclassifying diagnostic outcomes when compared to using survey data. Although the definition we used for homelessness has a low sensitivity, it has a very high specificity of 99.9%,^14^ and likely includes almost exclusively individuals who are experiencing homelessness. Finally, while the high prevalence of AMH among PEH is not a novel finding, few studies have characterised the range of specific AMH conditions in a Canadian population—and this is important for planning for policy, service delivery and other interventions.

## Conclusions

Given the high amount of healthcare utilisation and multimorbidity among PEH with AMH conditions, housing supports should integrate access to collaborative healthcare aiming to improve both mental and physical health. Integration of collaborative models of care with housing supports may improve future outcomes. These findings align with the provincial goals for mental health and addictions in Alberta, underscoring the importance of integrated healthcare and housing services to address the complex needs of PEH with AMH conditions. The next step for this research is to examine outcomes (e.g., overall mortality, death by suicide, suicide attempts, health encounters involving police and ED visits) for these individuals over a 5-year follow-up period using propensity score methods to account for the differences in baseline characteristics between PEH and those not experiencing homelessness.

## Supplemental Material

sj-docx-1-cpa-10.1177_07067437251380732 - Supplemental material for Characteristics of Adults With Addictions and Mental Health Problems Who Have Experienced Homelessness: A Population-Based Study From Alberta, Canada: Caractéristiques des adultes aux prises avec des problèmes de dépendance et de santé mentale et ayant connu l’itinérance : une étude fondée sur la population de l’Alberta, Canada Supplemental material, sj-docx-1-cpa-10.1177_07067437251380732 for Characteristics of Adults With Addictions and Mental Health Problems Who Have Experienced Homelessness: A Population-Based Study From Alberta, Canada: Caractéristiques des adultes aux prises avec des problèmes de dépendance et de santé mentale et ayant connu l’itinérance : une étude fondée sur la population de l’Alberta, Canada by Rebeca Barry, Geoffrey Messier, Anees Bahji, Gina Dimitropoulos, Sumantra Monty Ghosh, Julia Kirkham, Scott B Patten, Katherine Rittenbach, Faezehsadat Shahidi, David Tano, Valerie H Taylor and Dallas P Seitz in The Canadian Journal of Psychiatry
