# Supplementary material for: Characteristics of Adults With Addictions and Mental Health Problems Who Have Experienced Homelessness: A Population-Based Study From Alberta, Canada: Caractéristiques des adultes aux prises avec des problèmes de dépendance et de santé mentale et ayant connu l’itinérance : une étude fondée sur la population de l’Alberta, Canada
Source: Can J Psychiatry. 2025 Oct 8;71(2):139–48. doi: 10.1177/07067437251380732 (PMC12507784; doi:10.1177/07067437251380732)
Supplement: sj-docx-1-cpa-10.1177_07067437251380732 - Supplemental material for Characteristics of Adults With Addictions and Mental Health Problems Who Have Experienced Homelessness: A Population-Based Study From Alberta, Canada: Caractéristiques des adultes aux prises avec des problèmes de dépendance et de sa [file sj-docx-1-cpa-10.1177_07067437251380732.docx]

**Supplementary Tables**

eTable 1: AMH diagnosis codes

eTable 2: Healthcare Utilization Frequency

eTable 3: Baseline characteristics among People who did and did not Experience Homelessness (Sensitivity Analysis)

eTable 4: AMH Diagnoses in Past 5 years among People who did and did not Experience Homelessness (Sensitivity Analysis)

eTable 5: Number of AMH Categories at Baseline among People who did and did not Experience Homelessness (Sensitivity Analysis)

eTable 6: Healthcare Utilization among People who did and did not Experience Homelessness

**eTable 1: AMH diagnosis codes**

|  | **AMH Case Definitions** |
| --- | --- |
| **AMH Conditions** | MUST have one or more of the following to be included in the cohort: Substance use disorder, Mood disorder, Anxiety disorder, Psychotic disorder, Cognitive Disorders, Developmental disability, Personality disorder, Conduct, Disorder, Eating disorder, Sexual disorder, ADHD, Organic disorder, Sleep disorder, Somatic symptoms and related disorders, and/or deliberate self-harm |
| **Substance Use Disorder** | DAD, any hospitalization: F10.X—F19.X (except F17), F55.X, F63.X  OR  Physician Claims: 2 or more claims at least 30 days apart in 2-year period with diagnosis: 291, 292,303, 304, 305 (except 305.1) |
| **Mood disorder** | DAD: F30.X, F31.X, F32.X, F33.X, F34.X, F38.X, F39.X, F53.0  OR  2 physicians claims at least 30 days apart within a 2 year period with one or more of the following codes:  Physician Claims: 296, 311 |
| **Anxiety disorder** | DAD: F40.X, F41.X, F42.X, F43.X, F48.8, F48.9  OR  2 physicians claims at least 30 days apart within a 2 year period with one or more of the following codes:  Physician Claims: 300, 308, 309 |
| **Psychotic disorder** | DAD: F06.0, F06.1, F06.2, F06.0-2, F20.X, F22.X, F23.X, F24.X, F25.X, F26.X, F27.X, F28.X, F29.X, F22-F29, F53.1  OR  2 physicians claims at least 30 days apart within a 2 year period with one or more of the following codes:  Physician Claims: 295, 297, 298 |
| **Cognitive disorders** | DAD: F00X, F01.X, F02.X, F03.X G30.X  OR  2 physicians claims at least 30 days apart within a 2 year period with one or more of the following codes:  Physician Claims: 290, 331  OR  Prescription of dementia medication: Pharmacy Information Network: DINS for Donepezil, Galantamine, Rivastigmine or Memantine (Excel file attached) |
| **Developmental disability** | DAD: F70.0, F70.1, F70.8, F70.9, F71.0, F71.1, F71.8, F71.9, F72.0, F72.1, F72.8, F72.9, F73.0, F73.1, F73.8, F73.9, F78.0, F78.1, F78.8, F78.9, F79.0, F79.1, F79.8, F79.9, F84.0, F84.1, F84.3, F84.4, F84.5, F84.8, F84.9  OR  2 physicians claims at least 30 days apart within a 2 year period with one or more of the following codes:  Physician Claims: 299, 317, 318, 319 |
| **Personality disorders** | DAD: F21.X, F60.X, F61.X, F62.X, F68.1, F68.8, F69.X, F91.X  OR  2 physicians claims at least 30 days apart within a 2 year period with one or more of the following codes:  Physician Claims: 301, 312 |
| **Eating disorders** | DAD: F50  OR  2 physicians claims at least 30 days apart within a 2 year period with one or more of the following codes:  Physician Claims: 307.1, 307.51, 307.50 |
| **Sexual disorders** | DAD: F52.X, F64.X, F65.X  OR  2 physicians claims at least 30 days apart within a 2 year period with one or more of the following codes:  Physician Claims: 302 |
| **Other childhood and developmental disorders (not captured in first definition)** | DAD: F80.X, F81.X, F82.X, F83.X, F85.X, F86.X, F87.X, F88.X, F89.X. F92.X, F93.X, F94.X, F95.X, F96.X, F97.X, F98.X, F99.X  OR  2 physicians claims at least 30 days apart within a 2 year period with one or more of the following codes:  Physician Claims: 307.0, 307.2, 307.3, 307.52, 307.53, 307.54, 307.59, 307.6, 307.7, 307.8, 307.9, 313, 315. |
| **ADHD** | DAD: F90  OR  2 physicians claims at least 30 days apart within a 2 year period with one or more of the following codes:  Physician Claims: 314 |
| **Organic disorders** | DAD: F06.X, F07.X, F09.X, F59.X  OR  2 physicians claims at least 30 days apart within a 2 year period with one or more of the following codes:  Physician Claims: 293, 294, 310 |
| **Sleep disorders** | DAD: F51  OR  2 physicians claims at least 30 days apart within a 2 year period with one or more of the following codes:  Physician Claims: 307.4 |
| **Somatic symptoms and Related Disorders** | DAD: F44.X, F45.X, F48.0, F48.1, F54.X, F68.0  OR  2 physicians claims at least 30 days apart within a 2 year period with one or more of the following codes:  Physician Claims: 306, 316 |
| **Deliberate self-harm** | DAD: X60-X84, Y10-Y19, Y28 when DX10CODE1 is not equal to F06-F99 |

**eTable 2: Healthcare Utilization Frequency**

| Utilization | Experiencing Homelessness  (N=3,390)  N, (%) | Not Experiencing Homelessness  (N=659,224)  N, (%) | p-value |
| --- | --- | --- | --- |
| **Physician Visits (Claims)** |  |  |  |
| Number of Family Physician Visits (1 year) (Median, IQR) | 6 (3, 12) | 3 (2, 6) | <0.001 |
| Number of Family Physician Visits (5 year) (Median, IQR) | 24 (12, 44) | 17 (11, 26) | <0.001 |
| Number of Psychiatrist Visits (1 year) (Median, IQR) | 1 (0, 5) | 0 (0, 0) | <0.001 |
| Number of Psychiatrist Visits (5 year) (Median, IQR) | 4 (1, 14) | 0 (0, 1) | <0.001 |
| Number of Other Specialist visits (1 year) (Median, IQR) | 1 (0, 2) | 0 (0, 1) | <0.001 |
| Number of Other Specialist visits (5 year) (Median, IQR) | 3 (1, 6) | 3 (1, 7) | <0.001 |
| **Emergency Visits (NACRS)** |  |  |  |
| Number of Emergency Department Visits (1 year) (Median, IQR) | 6 (3, 11) | 1 (0, 2) | <0.001 |
| Number of Emergency Department Visits (5 year) (Median, IQR) | 17 (9, 33) | 3 (1, 7) | <0.001 |
| Number of Emergency Department Mental Health Visits (1 year) (Median, IQR) | 2 (0, 4) | 0 (0, 0) | <0.001 |
| Number of Emergency Department Mental Health Visits (5 year) (Median, IQR) | 4 (1, 9) | 0 (0, 0) | <0.001 |
| Number of non-Mental Health Emergency Visits (1 year) (Median, IQR) | 4 (2, 8) | 1 (0, 2) | <0.001 |
| Number of non-Mental Health Emergency Visits (5 year) (Median, IQR) | 12 (6, 25) | 3 (1, 6) | <0.001 |
| **Hospitalizations (DAD)** |  |  |  |
| Number of hospitalizations (1 year) (Median, IQR) | 1 (0, 2) | 0 (0, 0) | <0.001 |
| Number of hospitalizations (5 year) (Median, IQR) | 2 (1, 4) | 0 (0, 0) | <0.001 |

**eTable 3: Baseline characteristics among People who did and did not Experience Homelessness (Sensitivity Analysis)**

|  | Experiencing Homelessness (N=3,951)  N, (%) | Not Experiencing Homelessness (N=658,633)  N, (%) | Adjusted Odds Ratio (95% CI) |
| --- | --- | --- | --- |
| Sex (% Male) | 2,541 (64.3) | 266,566 (40.5) | 2.66 (2.49, 2.84) |
| Age (mean, SD) | 39.8 (12.2) | 41.6 (13.1) | - |
| 18 – 25, N (%)  26 – 35, N  36 – 45, N  46 – 55, N  56 – 65, N | 558 (14.1)  1,061 (26.9)  962 (24.4)  831 (21.0)  539 (13.6) | 90,477 (13.7)  149,890 (22.8)  148,596 (22.6)  143,833 (21.8)  125,867 (19.1) | REF  1.17 (1.06, 1.30)  1.06 (0.96. 1.18)  0.94 (0.85, 1.05)  0.69 (0.62, 0.79) |
| Pampalon Index: Social |  |  |  |
| 1  greater deprivation  2  3  4  5 | 193 (5.8)  198 (5.9)  358 (10.7)  766 (22.8)  1,841 (54.9) | 104,762 (16.6)  95,793 (15.9)  118,097 (18.7)  142,925 (22.7)  169,437 (26.9) | REF  1.12 (0.92, 1.37)  1.65 (1.38, 1.96)  2.90 (2.47, 3.40)  5.77 (4.98, 6.70) |
| Pampalon Index: Material |  |  |  |
| 1  greater deprivation  2  3  4  5 | 610 (18.2)  361 (10.8)  411 (12.3)  578 (17.2)  1,396 (41.6) | 121,098 (19.2)  117,492 (18.6)  122,917 (19.5)  130,132 (20.6)  139,375 (22.1) | REF  0.62 (0.60, 0.71)  0.67 (0.60, 0.76)  0.90 (0.80, 1.01)  2.00 (1.81, 2.19) |
| Health encounters involving police Past 5 years | 652 (16.5) | 5,309 (0.8) | 18.43 (16.83, 20.18) |

**eTable 4: AMH Diagnoses in Past 5 years among People who did and did not Experience Homelessness (Sensitivity Analysis)**

| Disorder | Experiencing Homelessness  (N=3,951)  N, (%) | Not Experiencing Homelessness  (N=658,633)  N, (%) | Adjusted* Odds Ratio (95%CI) | p-value |
| --- | --- | --- | --- | --- |
| Anxiety disorder | 2,997 (75.9) | 453,041 (68.8) | 1.66 (1.55, 1.79) | <0.001 |
| Mood disorder | 2,798 (70.8) | 318,971 (48.4) | 2.82 (2.63, 3.02) | <0.001 |
| Substance use disorder | 3,193 (80.8) | 73,779 (11.2) | 30.71 (28.32, 33.31) | <0.001 |
| Psychotic disorder | 1,215 (30.8) | 25,006 (3.8) | 9.85 (9.19, 10.56) | <0.001 |
| Cognitive disorder | 81 (2.1) | 5,103 (0.8) | 2.95 (2.36, 3.69) | <0.001 |
| Developmental disability | 126 (3.2) | 6,353 (1.0) | 2.67 (2.22, 3.20) | <0.001 |
| Personality disorder | 1,216 (30.8) | 23,109 (3.5) | 11.67 (10.88, 12.52) | <0.001 |
| Eating disorder | 19 (0.5) | 1,899 (0.3) | 2.42 (1.54, 3.82) | 0.025 |
| Sexual disorder | 59 (1.5) | 15,460 (2.4) | 0.44 (0.34, 0.57) | 0.0004 |
| Other childhood and developmental disorders (not captured in first definition) | 203 (5.1) | 9,410 (1.4) | 3.71 (3.19, 4.32) | <0.001 |
| Attention Deficit Hyperactivity Disorder (ADHD) | 356 (9.0) | 40,427 (6.1) | 1.19 (1.06, 1.33) | <0.001 |
| Organic disorders | 277 (7.0) | 9,255 (1.4) | 5.07 (4.48, 5.75) | <0.001 |
| Sleep disorders | 187 (4.7) | 299,983 (4.6) | 0.96 (0.83, 1.12) | 0.587 |
| Somatic symptoms and related disorders | 79 (2.0) | 7,320 (1.1) | 1.17 (1.06, 1.30) | <0.001 |
| Self-harm | 257 (6.5) | 4,933 (0.8) | 9.10 (7.99, 10.37) | <0.001 |

*adjusted for age and sex

**eTable 5: Number of AMH Categories at Baseline among People who did and did not Experience Homelessness (Sensitivity Analysis)**

| Disorder | Experiencing Homelessness  (N=3,951)  N, (%) | Not Experiencing Homelessness  (N=658,663)  N, (%) | p-value |
| --- | --- | --- | --- |
| One type of AMH disorder | 703 (17.8) | 405,467 (61.6) | <0.001 |
| Two types of AMH disorders | 688 (17.4) | 178,592 (27.1) | <0.001 |
| Three types of AMH disorders | 893 (22.6) | 49,883 (7.6) | <0.001 |
| Four or more types of AMH disorders | 1,667 (42.2) | 24,721 (3.8) | <0.001 |
| Median number of comorbid AMH conditions, IQR | 3 (2, 4) | 1 (1, 2) | <0.001 |

**eTable 6: Healthcare Utilization among People who did and did not Experience Homelessness**

| Utilization | Experiencing Homelessness  (N=3,951)  N, (%) | Not Experiencing Homelessness  (N=658,663)  <%) | Adjusted Odds Ratio (95% CI) |
| --- | --- | --- | --- |
| **Physician Visits (Claims)** |  |  |  |
| Any Family Physician Visit (1 year) | 3,178 (93.8) | 609,942 (92.5) | 1.56 (1.36, 1.79) |
| Any Family Physician visit (5 year) | Supressed (~100) | Supressed (~100) | Supressed |
| Any Psychiatrist visit (1 year) | 2,096 (61.8) | 96,293 (14.6) | 8.92 (8.32, 9.57) |
| Any Psychiatrist visit (5 year) | 2,653 (78.2) | 193,733 (29.4) | 8.12 (7.47, 8.80) |
| Any Other Specialist visit (1 year) | 1,705 (50.3) | 304,007 (46.1) | 1.42 (1.33, 1.52) |
| Any Other Specialist visit (5 year) | 2,730 (80.5) | 531,104 (80.6) | 1.27 (1.16, 1.38) |
| **Emergency Visits (NACRS)** |  |  |  |
| Any Emergency Department visit (1 year) | 3,349 (98.8) | 360,069 (54.6) | 75.55 (52.25, 104.16) |
| Any Emergency Department visit (5 year) | SUP (~100) | 587,979 (89.2) | 158.54 (51.24, 490.5) |
| Any Emergency Department Mental Health visit (1 year) | 2,487 (73.4) | 49,035 (7.4) | 33.58 (31.07, 36.29) |
| Any Emergency Department Mental Health visit (5 year) | 2,975 (87.8) | 142,870 (21.7) | 25.44 (22.94, 28.21) |
| Any non-Mental Health Emergency Visits (1 year) | 3,083 (90.9) | 343,139 (52.1) | 10.63 (9.45, 11.96) |
| Any non-Mental Health Emergency Visits (5 year) | 3,343 (98.6) | 578,075 (87.7) | 11.85 (8.89, 15.81) |
| **Hospitalizations (DAD)** |  |  |  |
| Any hospitalization (1 year) | 2,200 (55.7) | 33,427 (5.1) | 23.08 (21.65, 24.61) |
| Any hospitalization (5 year) | 3,018 (76.4) | 116,427 (17.7) | 14.78 (13.72, 15.91) |
| Any mental health hospitalization (1 year) | 984 (24.9) | 8,314 (1.3) | 23.96 (22.19, 25.88) |
| Any mental health hospitalization (5 year) | 1,484 (37.6) | 30,644 (4.7) | 11.52 (10.78, 12.32) |
